# Hidden giant calyceal diverticulum: a rare case report caused by kidney stone obstruction of the calyceal neck

**DOI:** 10.1097/RC9.0000000000000771

**Published:** 2026-08-21

**Authors:** Wenbin Bao, Yangwen-Yi Liu, Jincui Yang, Xuebing Ma

**Affiliations:** a Department of Urology, The Fourth Affiliated Hospital of Dali University, Chuxiong, Yunnan, China; b Department of Diagnostic Services, Chuxiong People’s Hospital, Chuxiong, Yunnan, China

**Keywords:** calyceal diverticulum, case report, kidney stone, misdiagnosis, renal cyst

## Abstract

**Introduction and importance:**

A calyceal diverticulum is a rare cystic cavity that communicates with the renal calyx. Giant forms (>8 cm) are easily misdiagnosed as renal cysts. Unlike prior reports with patent diverticular necks on delayed imaging, this case features complete stone obstruction leading to false-negative conventional imaging, with the diagnosis revealed only after ureteral stenting and stone treatment.

**Case presentation:**

A 69-year-old man was incidentally found to have a 9 cm left renal cystic lesion. Initial CT/MRI suggested a simple cyst. After ureteral stent placement for an obstructing stone, the cyst shrank and its wall thickened. Retrograde flexible ureteroscopic laser lithotripsy fragmented an obstructing stone at the calyceal neck. Postoperative CT showed collapse of the cyst with gas and fluid accumulation, confirming a giant calyceal diverticulum that had decompressed after stone removal.

**Clinical discussion:**

The key diagnostic clue was dynamic reduction in size following ureteral stent placement – something impossible for a simple cyst. Complete obstruction of the diverticular neck by a stone caused false-negative findings on contrast studies. Treatment focused on stone removal, leading to diverticular collapse.

**Conclusion:**

For cystic lesions near the calyces with ipsilateral calculi, a calyceal diverticulum should be suspected even when contrast imaging is negative. Dynamic changes after ureteral stenting or stone intervention can aid in diagnosis and prevent unnecessary surgery.

## Introduction

A calyceal diverticulum is a rare, benign lesion lined by transitional epithelium and connected to the renal calyx via a narrow neck. Large diverticula are easily confused with simple renal cysts. Conventional non-contrast CT often fails to identify the communication, leading to misdiagnosis. Misdiagnosis and subsequent laparoscopic decortication without addressing the neck can cause urinary leakage or perirenal infection, requiring reoperation.

The novelty of this case lies in the complete obstruction of the diverticular neck by a stone, rendering all imaging (including delayed-contrast CT and MRI) falsely negative – a scenario rarely emphasized. In most prior reports, diagnosis by CT urography or retrograde pyelography assumed a patent neck. Here, dynamic changes after ureteral stenting and lithotripsy served as diagnostic tools. We report a giant (9 cm) calyceal diverticulum caused by stone obstruction, with the aim of improving recognition and reducing mistreatment. This work has been reported in accordance with the SCARE 2025 criteria (the SCARE checklist is provided as a supplementary file).

HIGHLIGHTS

- A giant calyceal diverticulum (9 cm) is extremely rare and mimics a renal cyst.
- Stone obstruction of the diverticular neck prevents contrast opacification on CT.
- Placement of a ureteral stent caused unexpected shrinkage of the cystic lesion.
- Stone fragmentation led to diverticular collapse, confirming the diagnosis.
- A high index of suspicion for a diverticulum is needed in pericalyceal cysts with stones.

## Case presentation

**Figure 1.**
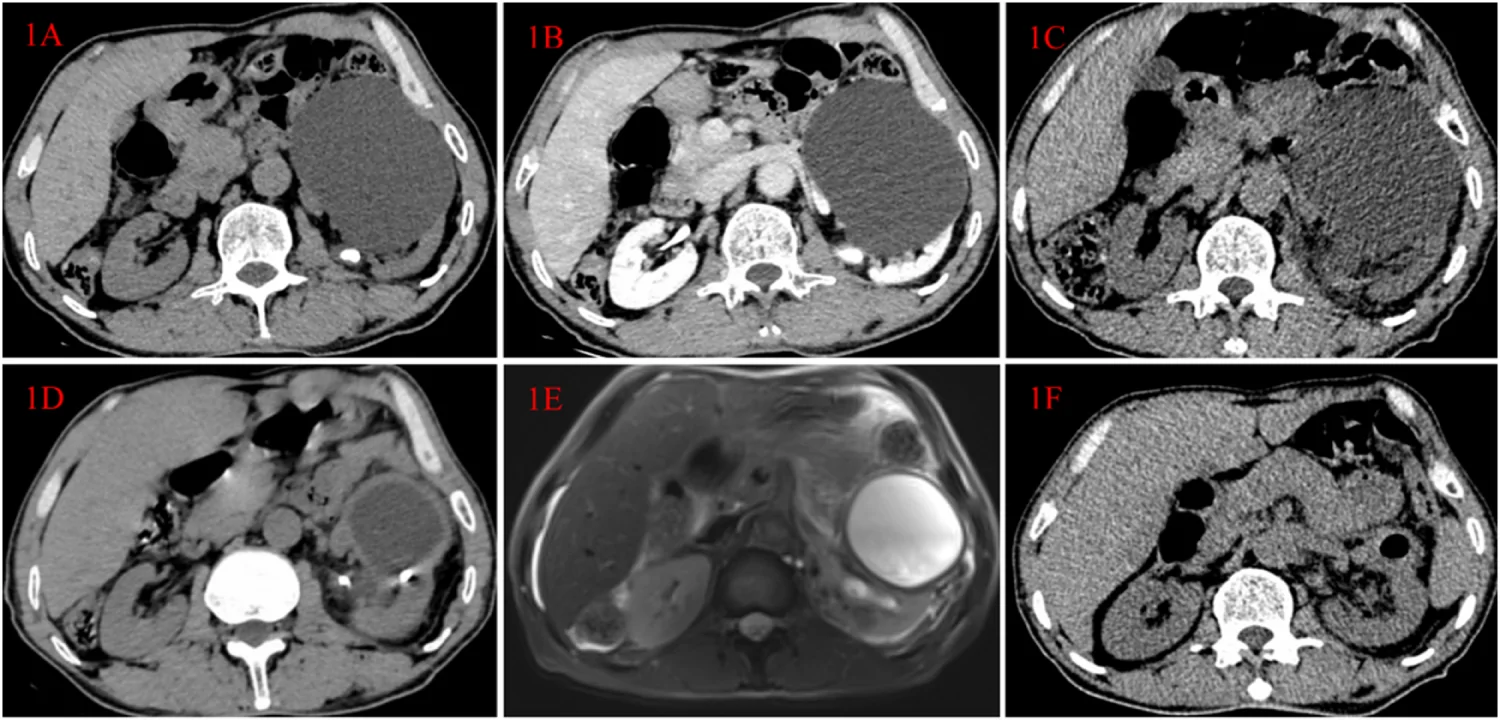
(A) On 13 November 2025, non-contrast CT on first admission showed a left renal cyst (92 × 71 mm) and a left renal calculus (10 mm). (B) Contrast-enhanced CT revealed no obvious enhancement of the lesion. (C) On 27 November 2025, the patient was admitted with renal colic. Urinary CT showed the left renal cyst with no significant change in size compared with the first admission. (D) On 5 January 2026, the patient was readmitted for flexible ureteroscopic lithotripsy. Urinary CT demonstrated that the left renal cyst had decreased in size, with a thickened wall suggestive of an infection. (E) For further characterization, MRI confirmed the presence of a left renal cyst. (F) On 1 February 2026, the patient returned for ureteral stent removal. Urinary CT showed a slight low-density lesion (18 mm) with gas accumulation in the left kidney.

A 69-year-old male presented with dysuria. Cystoscopy confirmed a urethral stricture. Initial non-contrast and contrast-enhanced CT scans showed: (1) a 92 × 71 mm oval, low-density lesion in the left kidney with no enhancement; (2) a 10 mm left renal calyceal stone (Fig. 1A and B). Laboratory findings: serum creatinine was 87 µmol/L (normal); urinalysis showed marked hematuria (RBC 5079/µL) with a normal WBC count and a negative culture. The initial diagnosis was a renal cyst. Laparoscopic decortication was planned.

On 27 November 2025, he presented with a 15-hour history of left flank pain. CT revealed a left ureteral stone with mild hydronephrosis and the same 92 × 71 mm cystic lesion (Fig. 1C). Laboratory results: serum creatinine, 129 µmol/L (mildly elevated); urinalysis: RBC, 3130/µL; WBC, 79.4/µL; culture negative. Transurethral left ureteral stent placement was performed on 2 December 2025; a tortuous upper ureter precluded immediate lithotripsy.

**Figure 2.**
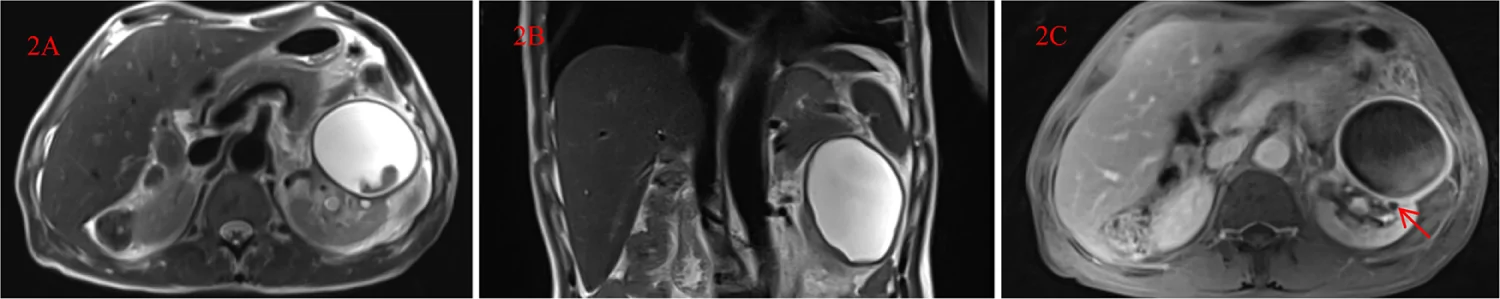
(A) Axial T2-weighted MRI shows a cystic lesion in the left kidney, measuring approximately 6.9 cm × 6.2 cm × 8.1 cm, with a slightly thickened wall. (B) Coronal T2-weighted MRI. (C) Dynamic contrast-enhanced MRI shows moderate enhancement of the cyst wall, with a calculus visible (indicated by the red arrow).

On 4 January 2026, he was readmitted for stone management. Laboratory results: serum creatinine, 135 µmol/L; urinalysis: RBCs, 2211/µL; WBCs, 28,563/µL; urine culture grew E. coli. Preoperative CT showed the stent in place, reduced hydronephrosis, and shrinkage of the cystic lesion to 80 × 56 × 47 mm with wall thickening (Fig. 1D). MRI confirmed multiple cystic lesions, the largest measuring 6.9 × 6.2 × 8.1 cm, with wall enhancement (Fig. 2). On 12 January 2026, left flexible ureteroscopic laser lithotripsy was performed. Intraoperatively, a 0.6 × 0.8 cm stone was found in the lower calyx. Stone impaction at the calyceal neck was observed, as supported by CT showing lesion reduction. No intraoperative images were captured – a limitation.

On 1 February 2026, a pre-stent-removal CT scan showed the original cystic lesion had been replaced by mild low-density changes with gas accumulation, suggestive of an abscess or perirenal infection (Fig. 1F). The giant lesion had markedly decreased in size and was replaced by inflammatory changes.

### Final diagnosis

Based on the history and dynamic imaging, the final diagnosis is left calyceal diverticulum – extreme dilatation caused by obstruction of the neck by a stone, followed by diverticular collapse after stone displacement, with secondary infection.

### Treatment and outcome

Antibiotics (ceftazidime 1 g BID for 3 days) were given. On 5 February 2026, the stent was removed cystoscopically. The patient recovered well. Follow-up at 3 months (5 April 2026) showed complete resolution of the fluid collection, no recurrence, no residual stone, and a normal creatinine level (80 µmol/L).

## Discussion

Calyceal diverticulum is a relatively rare lesion, with an incidence of 0.21%–0.45%, mostly congenital, with a minority caused by obstruction, infection, or trauma. It has no gender or age predilection and is usually detected incidentally by intravenous urography (IVU)[1]. Approximately 50% of patients present with clinical symptoms such as low back pain, urinary tract infection, and hematuria, while the remainder are asymptomatic[2].

The key challenge in diagnosing a calyceal diverticulum is differentiating it from simple renal cysts, as clinical symptoms and signs are non-specific. Imaging examinations are the mainstay of diagnosis. Contrast-enhanced CT with delayed scanning is recommended to improve the preoperative diagnostic rate, as prolonged scanning time increases the probability of the contrast agent entering the diverticulum, and the secretory function of the transitional epithelial lining also facilitates diverticular visualization[3]. However, false negatives may occur even with delayed scanning, especially when the diverticular neck is completely obstructed[4], as seen in our case.

Alternative imaging modalities can be used as supplements. For example, Wang *et al*[5] first reported the diagnosis of calyceal diverticulum using 18 F-FDG-PET scanning. Intraoperatively, several methods can help differentiate calyceal diverticulum from renal cysts: qualitative protein testing of cyst fluid (positive for simple renal cysts due to protein content, negative for diverticular urine)[6]; quantitative creatinine measurement (diverticular urine creatinine is significantly higher than serum creatinine)[7]; and observation of clear fluid outflow from the cyst cavity after intravenous furosemide injection and reduction of pneumoperitoneum pressure[8].

Misdiagnosis of a calyceal diverticulum as a simple renal cyst can lead to severe postoperative complications. In our case, the initial misdiagnosis was attributed to the absence of typical imaging features on conventional CT: the narrow diverticular neck was completely occluded by the stone, and the patient had no obvious lumbago at the initial visit. The key turning point was the dynamic imaging changes after ureteral stent placement: the cystic lesion shrank significantly and the wall thickened, findings inconsistent with a simple renal cyst (which would not change in size with stent placement). We hypothesize that stent placement reduced intrapelvic pressure, leading to pressure-dependent microleakage or partial reabsorption of diverticular urine, resulting in decreased intracavitary pressure, shrinkage, and wall thickening.

The definitive diagnosis was confirmed by interventional treatment of the calculus: after fragmentation of the obstructing stone, the diverticular neck reopened, urine drained rapidly, and the diverticulum collapsed. A postoperative CT scan showed localized fluid and gas accumulation, consistent with infection following drainage. This dynamic process further supported the diagnosis.

Regarding treatment, simple calyceal diverticula are often asymptomatic or present with mild symptoms. Those with a diameter <2.5 cm can be managed conservatively with regular follow-up. Surgical treatment should be considered in patients presenting with symptoms such as low back pain, fever, hematuria, poor drainage due to urine reflux into the diverticulum, or secondary complications such as stone formation, infection, or tumors, as well as in those with a diverticulum diameter >2.5 cm. Currently, minimally invasive laparoscopic techniques are rapidly evolving. Open surgery is rarely used due to its significant trauma, but it still has considerable value in cases that are difficult to manage with minimally invasive approaches. Literature reports indicate that robot-assisted laparoscopic calyceal diverticulectomy achieves satisfactory outcomes with less trauma and greater safety[9]. Diverticula located on the dorsal side of the kidney are suitable for percutaneous nephrolithotomy, whereas those on the ventral side or in the lower pole are suitable for laparoscopic surgery[10]. For large diverticula that cause severe renal damage, nephrectomy may be performed; for smaller diverticula, nephron-sparing procedures such as diverticulectomy, partial nephrectomy, or wedge resection are options. In this case, the calyceal diverticulum was considered to be caused by a stone obstructing the calyceal neck; therefore, the mainstay of treatment was stone management, and after stone treatment, the diverticulum gradually shrank.

Limitations: No intraoperative photographs or videos were captured. Delayed contrast-enhanced CT (≥1 hour) with three-dimensional imaging was not performed because the patient presented with renal colic; this might have provided additional information if the neck had not been completely obstructed. The follow-up duration is only 3 months; longer follow-up is needed to assess for diverticular or stone recurrence.

## Conclusion

The clinical implication of this case is that clinicians should maintain a high index of suspicion for calyceal diverticulum when evaluating a cystic lesion around the renal calyces, especially with ipsilateral renal calculi. For suspected cases with negative contrast-enhanced CT, delayed scanning at 1–2 hours is recommended. MRI or contrast-enhanced CT can help evaluate for cyst wall enhancement. Additionally, treatment of concurrent stones can serve as a diagnostic test, as seen in our case. Selecting appropriate imaging modalities based on individual patient conditions is crucial for accurate diagnosis and avoiding unnecessary surgery.

## Data Availability

All data generated or analyzed during this study are included in this published article and its supplementary information files.

## Patient perspective

The patient expressed relief at avoiding unnecessary surgery and gratitude for the careful follow-up.
